# The value of spectral CT quantitative parameters in predicting Ki-67 level in ovarian cancer

**DOI:** 10.3389/fonc.2025.1576690

**Published:** 2025-05-14

**Authors:** Siwen Pang, Meng Wu, Haijia Yu, Tiantian Ma, Jianhua Liu, Siwen Liu

**Affiliations:** Department of Radiology, Second Affiliated Hospital of Jilin University, Changchun, China

**Keywords:** ovarian cancer, Ki-67 level, quantitative parameters, iodine concentration, effective atomic number, the slope of the spectral curve, spectral CT

## Abstract

**Objective:**

The objective of this study is to evaluate the predictive value of quantitative parameters from spectral CT for Ki-67 expression in ovarian cancer (OC).

**Methods:**

Spectral CT imaging data from 39 patients with ovarian cancer by pathology, encompassing 52 lesions overall, were collected retrospectively and split into two groups based on immunohistochemical results. Tumor solid components in arterial, venous, and delayed phases can be measured using post-processing software to obtain the quantitative parameters of spectral CT. An independent sample t-test was implemented for evaluating spectral CT parameters between two groups, and a Spearman correlation coefficient was applied among all participants to estimate the relationship between spectral parameters and Ki-67 levels. Moreover, an examination of the receiver operating characteristic (ROC) curve was conducted to assess the diagnostic efficacy of the significantly different parameters between the two groups.

**Results:**

The Ki-67 high-level group includes 22 patients and 29 lesions, while the Ki-67 low-level group contains 17 patients and 23 lesions. The A-sIC, A-sZeff, V-IC, D-Zeff, and D-sZeff values in the Ki-67 high-level group were greater than those in the Ki-67 low-level group (*P* =0.028, AUC = 0.705; *P* < 0.001, AUC = 0.742; *P* = 0.047, AUC = 0.657; *P* = 0.014, AUC = 0.665; and *P* = 0.006, AUC = 0.675, respectively). For correlation analysis, A-IC, A-sIC, A-Zeff, A-sZeff, A-λHU, D-IC, D-Zeff, and D-sZeff were positively correlated with Ki-67 levels, with correlation coefficients ranging from 0.277 to 0.417, *P*<0.05. Through multiple logistic regression, the combined model that included 5 quantitative parameters showed the highest diagnostic performance, with a sensitivity of 93.10%, a specificity of 60.90%, and an AUC value of 0.808.

**Conclusion:**

Spectral CT provides multi-parametric imaging data and is useful in predicting Ki-67 expression in ovarian cancer, delivering comprehensive and reliable imaging evidence for the formulation of therapeutic treatment options.

## 1 Introduction

Ovarian cancer (OC) is a prevalent malignant tumor in gynecology, and its mortality rate rises year after year, making it one of the leading causes of cancer death and posing a major threat to patients’ lives (1, 2). Because of the non-specific clinical symptoms of ovarian cancer, the majority of patients are detected for the first time in stages III and IV, resulting in an extremely low survival rate. In contrast, a survival probability of over 90% can be achieved through early diagnosis (3, 4). In clinical practice, certain indicators such as CA125 and CA199 are closely related to the occurrence of ovarian cancer, and an increase in these indicators in patients often indicates the emergence of ovarian cancer. Any histological component can become the site of ovarian cancer, and the most common kind is high-grade serous carcinoma that starts in the epithelium (5). Although surgery and chemotherapy are the most common treatments for epithelial ovarian cancer, individuals with advanced epithelial ovarian cancer still have a poor prognosis, and more research is needed to develop improved diagnostic and therapeutic approaches.

Numerous factors, such as the tumor’s stage, differentiation grade, and histological type, affect the prognosis of individuals with ovarian cancer (5, 6). Ki-67, an essential proliferation marker, is frequently detected in several types of malignant tumors and can be used to assess cell growth scores (7). Numerous studies (8, 9) have indicated that the level of Ki-67 is typically connected to the tumor’s risk of malignancy and recurrence and might be a helpful indicator for determining a patient’s prognosis. Furthermore, research has shown that Ki-67 has advantages in discriminating between benign and malignant cancers, identifying tumor subtypes, and guiding chemotherapy decisions, making it an essential signal for disease diagnosis, treatment, and prognosis (10–12).

Multiple histological types abound in ovarian tumors, and their endocrine action and tissue composition are complicated, leading to various imaging appearances. Ovarian cancer is currently diagnosed using a variety of imaging methods, such as ultrasound, multi detector computed tomography (MDCT), and magnetic resonance imaging (MRI). Among them, ultrasound is frequently employed to screen for ovarian cancer; additionally, MDCT and MRI provide specific advantages for the detection and staging of ovarian cancer (13, 14). Nevertheless, spectral CT, an emerging imaging technology, is capable of not only qualitatively diagnosing lesions based on morphology but also quantitatively analyzing the condition of lesions through multiple parameters, thereby providing patients with a greater variety of imaging information. Spectral CT is frequently utilized in disease detection because it serves benefits over traditional CT, including the capacity to separate materials, low-dose imaging, artifact reduction, functional imaging, and multi-parameter analysis. Furthermore, spectral CT characteristics can offer more useful imaging evidence for clinical practice and are frequently employed in oncology (15, 16). According to several research, spectral CT is extensively utilized in the diagnosis of ovarian cancer; it offers notable benefits for the differential detection of benign and malignant cancers (17, 18).

The development of a variety of malignancies, particularly colon cancer, ovarian cancer, and breast cancer, is closely associated with Ki-67 (9, 19, 20). Moreover, some studies (9, 21)have demonstrated that the level of Ki-67 is linked to the prognosis and subsequent treatment. However, no studies have utilized quantitative parameters of spectral CT to assess the level of Ki-67 in ovarian cancer patients. The efficacy of spectral CT for evaluating Ki-67 level in ovarian cancer is not yet established. Consequently, this investigation implemented numerous spectral CT parameters to estimate Ki-67 levels in ovarian cancer and extend patients’ imaging information.

## 2 Methods

### 2.1 Patient characteristics

**Figure 1:**
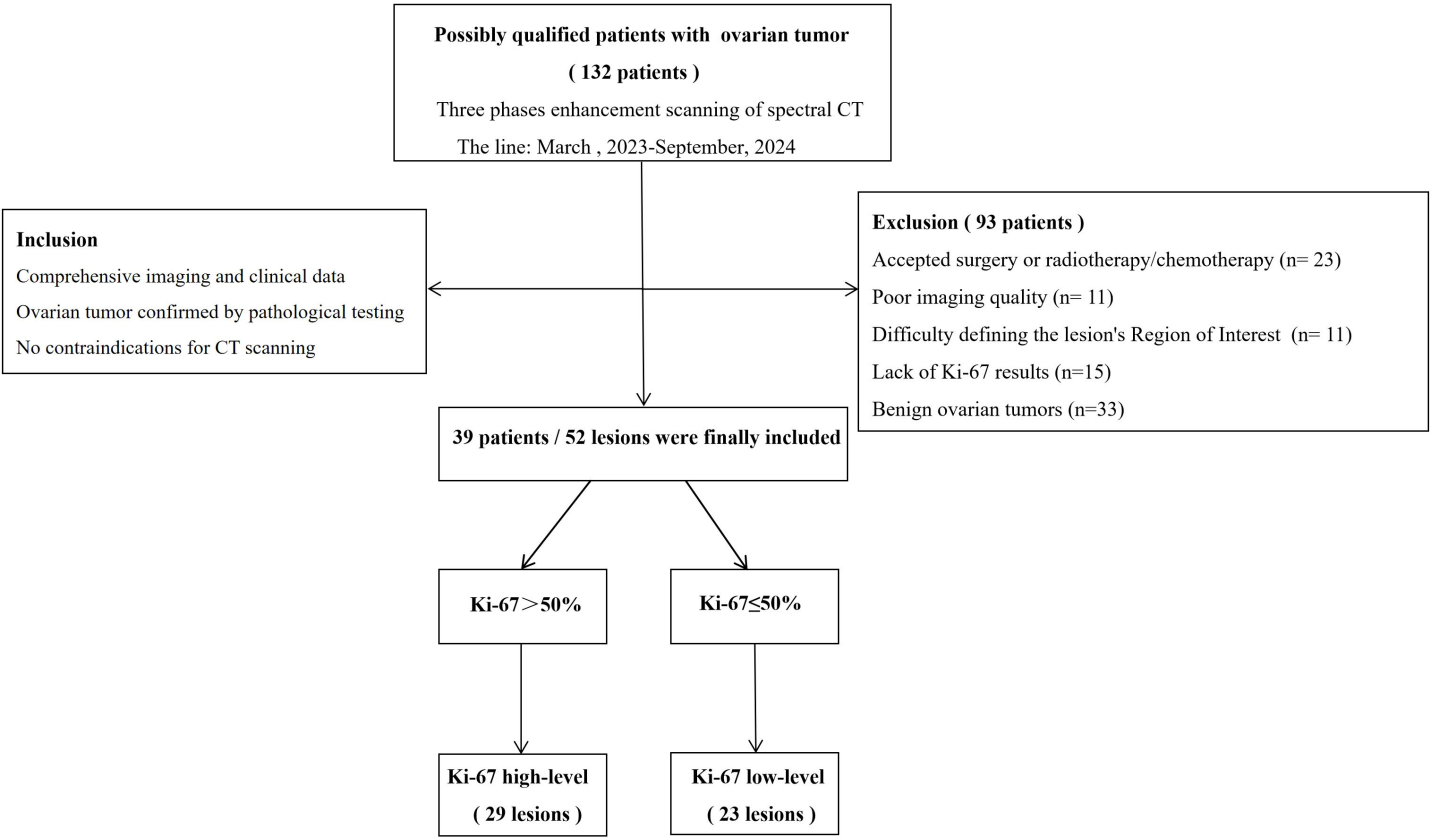
Process diagram for selecting patients.

The Hospital Ethics Committee has approved this study based on exemptions covering the signing of informed consent forms, and all procedures comply with applicable regulations. From March 2023 to September 2024, spectral CT scans were performed on 132 patients suspected of having ovarian cancer in clinical practice. Inclusion criteria: (1) comprehensive imaging and clinical data; (2) ovarian tumor confirmed by pathological testing; (3) no contraindications for CT scanning. For the following reasons, a total of 93 cases were not included: (1) accepted surgery or radiotherapy/chemotherapy (n = 23); (2) poor imaging quality (n = 11); (3) difficulty defining the lesion’s Region of Interest (ROI) (n = 11); (4) lack of Ki-67 results (n = 15), and (5) benign ovarian tumors (n = 33). Finally, 39 instances with 52 ovarian tumor lesions were the last patients to be enrolled. **Figure 1** shows the patient selection process.

### 2.2 Spectral CT protocol

Two weeks elapsed between the scan and the surgery. Before going through the scanning process, patients had to refrain from consuming anything for at least eight hours. Additionally, at least 800 ml of water should be drunk in order to maximize stomach fullness and improve lesion visibility. A 256-row CT scanner (GE Revolution CT) was employed to acquire plain and three-phase enhanced scan images, with a scanning range of the diaphragm to the pubic symphysis. The following are the specific parameters: The tube voltage quickly switches between 80 kVp/140 kVp; tube current: 445 mA; scanning thickness: 5 mm; reconstruction thickness: 1.25 mm; pitch: 0.6; rotational time: 0.5s/r; matrix: 512 × 512. Using a high-pressure injector (Stellant, Medrad, Byer HealthCare), 70 mL of iodine contrast agent was administered at a rate of 5 mL/s through the central elbow vein. Next, delivered 40 mL of physiological saline. The bolus tracking method was used to acquire enhanced images of the arterial, venous, and delayed phases. Arterial phase (AP), venous phase (VP), and delayed phase (DP) scan delays were set at 30 seconds, 55 seconds, and 120 seconds, respectively.

### 2.3 Image post-processing and analysis

The initial spectral images were imported to the GE AW4.7 workstation, and image post-processing was performed using the GSI Viewer program. Two radiologists with over a decade of experience and a specialization in gynaecology assessed the image condition to guarantee the accuracy of image processing. To avoid prejudice, they were blinded to the pathology results. Any discrepancies that had surfaced were discussed between the two radiologists. For optimal precision, the subsequent guidelines were implemented during region of interest (ROI) delineation: (1) The region with the most obvious enhancement of the solid part of the lesion on three adjacent layers was selected as the ROI for measurement, and all data were taken as the average of three measurements; (2)The delineation of ROI depends on the size of the lesion, avoiding cystic and hemorrhagic necrotic areas, and must be placed on the solid part of the lesion while controlling the size of ROI to be about 1/2-2/3 of the lesion; (3) The ROI replication function of the post-processing workstation ensures consistency in ROI size and position for each phase. By choosing iodine-based substances for substance separation in the three-phase energy spectrum enhanced image, the iodine concentration (IC) value and effective atomic number (Zeff) value within the lesion ROI of the three phases were recorded, along with CT values at 40 and 80 KeV. In addition, by plotting the spectral CT curve to calculate the slope of the curve (λHU) value, λHU = CT_40KeV_-CT_80keV_/40. Meanwhile, two associated parameters were assessed: sIC = IC_lesion_/IC_iliac artery_, sZeff = Zeff_lesion_/Zeff_iliac artery_. Each patient was split into two groups depending on the expression level of Ki-67 immunohistochemistry. Patients with Ki-67 values > 50% were categorized in the high-level group, whilst those with Ki-67 values ≤ 50% were placed in the low-level group (21, 22).

### 2.4 Statistical analysis

The statistical analysis was carried out employing IBM SPSS 27 (IBM, Armonk, NY, USA) and Graphpad Prism 10.2.3 (GraphPad Software, La Jolla, CA, USA). The intraclass correlation coefficient (ICC) was applied for consistency testing. ICCs above 0.80 were deemed highly reliable. The Shapiro-Wilk test validated the standard of the data distribution. The independent sample t-test was implemented to analyze the variations in spectral CT parameters and Ki-67 levels. The correlation between the spectral characteristics and the Ki-67 levels was evaluated using Spearman’s correlation coefficient. While categorical variables were stated in frequency (rate), the formula for continuous data was mean ± standard deviation. Receiver operating characteristic (ROC) curves were generated for parameters showing statistically differentials in order to evaluate diagnostic efficacy. In addition, the area under the curve (AUC) value, cut-off value, sensitivity, and specificity were calculated. The diagnostic efficacy of the ROC curves was compared by the Delong test. Single-factor and multiple-factor logistic regression analysis were used to evaluate the independent predictive factors for Ki-67. P value < 0.05 is considered statistically significant.

## 3 Results

### 3.1 Clinical characteristics of patients

**Figure 2:**
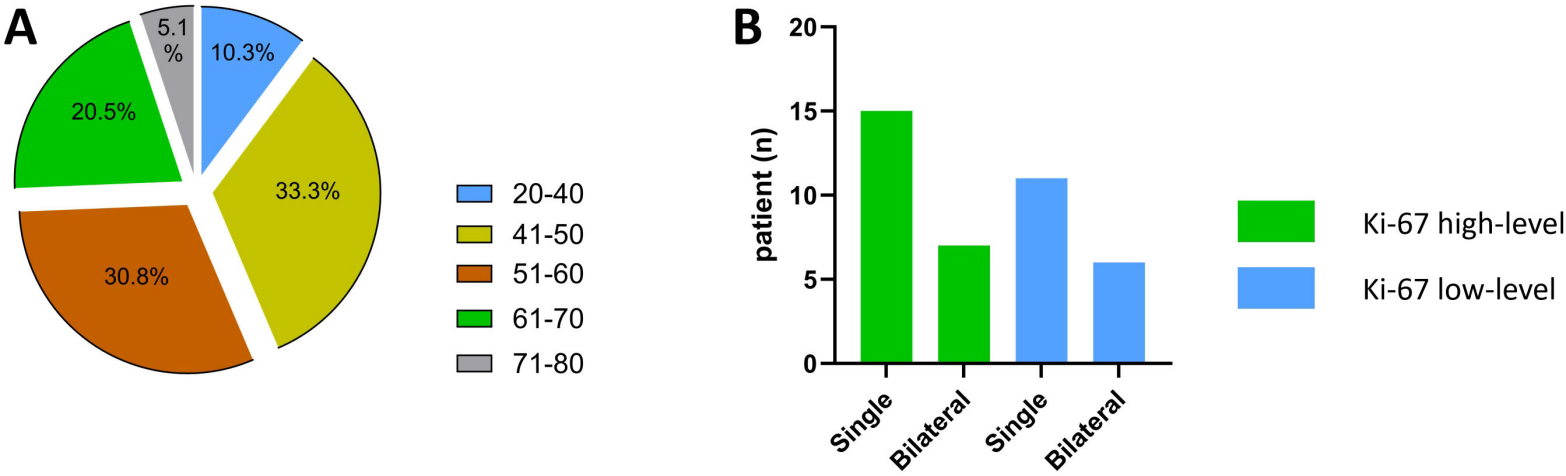
Patient age and lesion Single/Bilateral status. **(A)** Patient age status; **(B)** Lesion Single/Bilateral status.

This study included 39 participants with 52 lesions overall (average age: 53.69 years, range: 26–76 years). Age and the number of lesions did not significantly differ between the two groups (*P*=0.107, 0.819). In the Ki-67 high-level group (51.00 ± 10.25 years), there were 15 patients (68.2%) with single lesions and 7 patients (31.8%) with bilateral lesions; in the low-level group (57.18 ± 13.14 years), there were 11 patients (64.7%) with single lesions and 6 patients (35.3%) with bilateral lesions. Additionally, the two groups’ intratumoral bleeding, CA199 value, and ascites status varied not statistically meaningfully (*P*=0.582, 0.872, and 0.709, respectively). Nonetheless, the Ki-67 high-level group had greater values for CA125, and ROMA (premenopausal and postmenopausal) than the low-level group (*P* = 0.008, 0.022, and <0.001, respectively). **Table 1** and **Figure 2** display the pertinent clinical data.

**Table 1:** Clinical features of patients.

Characteristics	Ki-67 high-level (n=22)	Ki-67 low-level (n=17)	*P* Value
Age (years)	51.00 ± 10.25	57.18 ± 13.14	0.107
Single/Bilateral			0.819
Single	15 (15/22;68.2%)	11 (11/17;64.7%)	
Bilateral	7 (7/22;31.8%)	6 (6/17;35.3%)	
Intratumoral bleeding			0.582
Yes	4 (4/22;18.2%)	2 (2/17;11.8%)	
No	18 (18/22;81.8%)	15 (15/17;88.2%)	
CA125			0.008*
High	19 (19/22;86.4%)	8 (8/17;47.1%)	
Normal	3 (3/22;13.6%)	9 (9/17;52.9%)	
CA199			0.872
High	7 (7/22;31.8%)	5 (5/17;29.4%)	
Normal	15 (15/22;68.2%)	12 (12/17;70.6%)	
Premenopausal ROMA value			0.022*
High	18 (18/22;81.8%)	8 (8/17;47.1%)	
Normal	4 (4/22;18.2%)	9 (9/17;52.9%)	
Postmenopausal ROMA value			<0.001*
High	20 (20/22;90.9%)	7 (7/17;41.2%)	
Normal	2 (2/22;9.1%)	10 (10/17;58.8%)	
Ascites			0.709
Yes	2 (2/22;9.1%)	1 (1/17;5.9%)	
No	20 (20/22;90.9%)	16 (16/17;94.1%)	

*indicates the significant difference between two parameters.

### 3.2 Consistency in the measurement of spectral CT parameters

The spectral CT quantitative parameter values (IC, Zeff, λHU) of the three phases evaluated showed high consistency (ICC:0.943-0.990), with ICC values all above 0.80; the specific values are presented in **Table 2**.

**Table 2:** Consistency across readers for spectral CT parameters measurement.

Parameters	ICC	95%CI
A-IC	0.980	0.969-0.988
A-Zeff	0.957	0.933-0.974
A-λHU	0.972	0.956-0.983
V-IC	0.981	0.969-0.988
V-Zeff	0.943	0.912-0.965
V-λHU	0.965	0.946-0.979
D-IC	0.971	0.955-0.982
D-Zeff	0.990	0.984-0.994
D-λHU	0.972	0.956-0.983

ICC, Intraclass correlation coefficient; IC, Iodine concentration; Zeff, Effective atomic number; λHU, The slope of the spectral curve; A, Arterial phase; V, Venous phase; D, Delayed phase; 95%CI, 95% Confidence interval.

### 3.3 Comparison of spectral CT parameters

**Figure 3:**
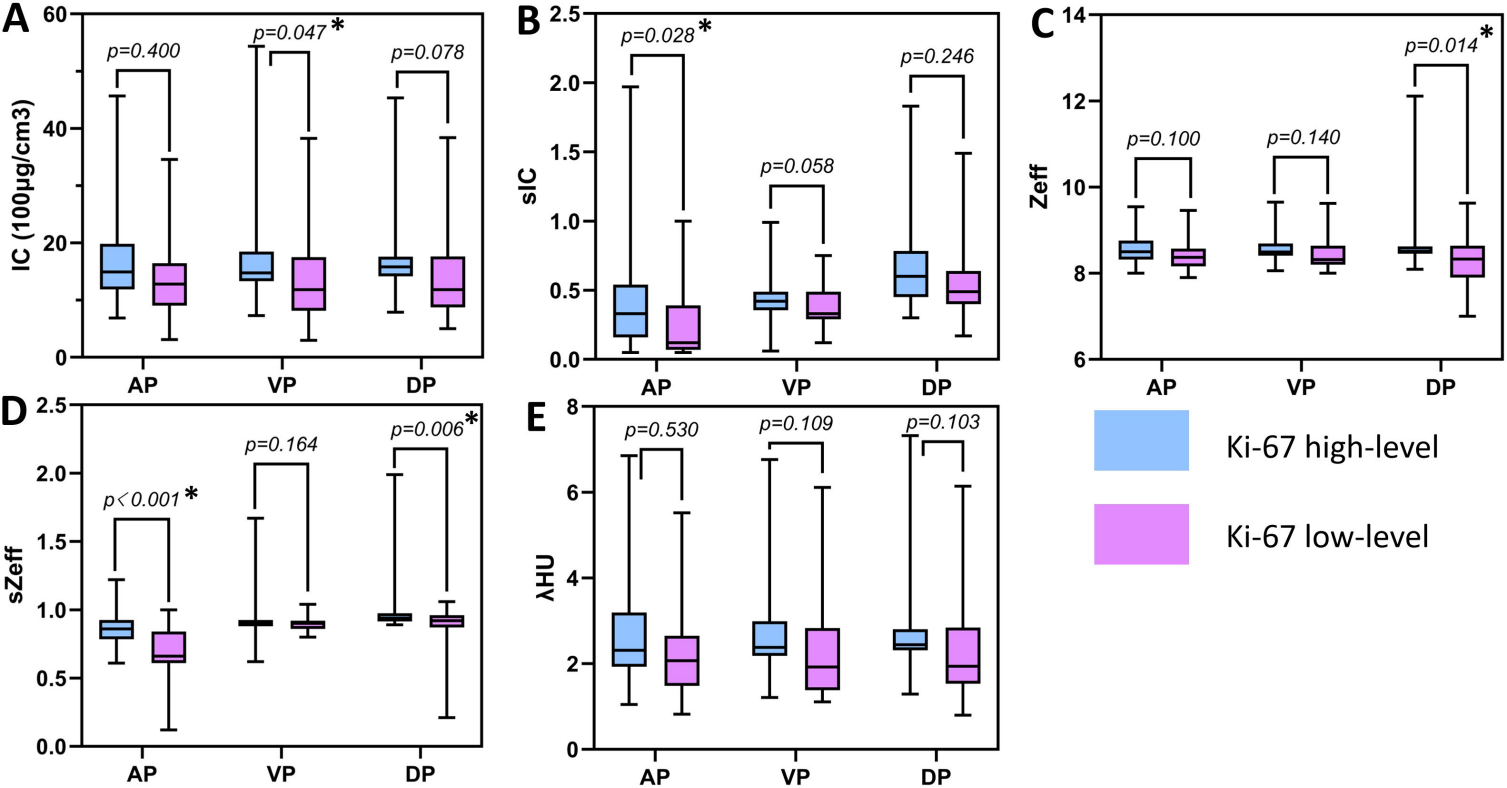
Boxplots of spectral CT parameters between two groups. **(A)** The IC value between two groups; **(B)** The sIC value between two groups; **(C)** The Zeff value between two groups; **(D)** The sZeff value between two groups; **(E)** The λHU value between two groups. * indicates the significant difference between two parameters.

**Figure 4:**
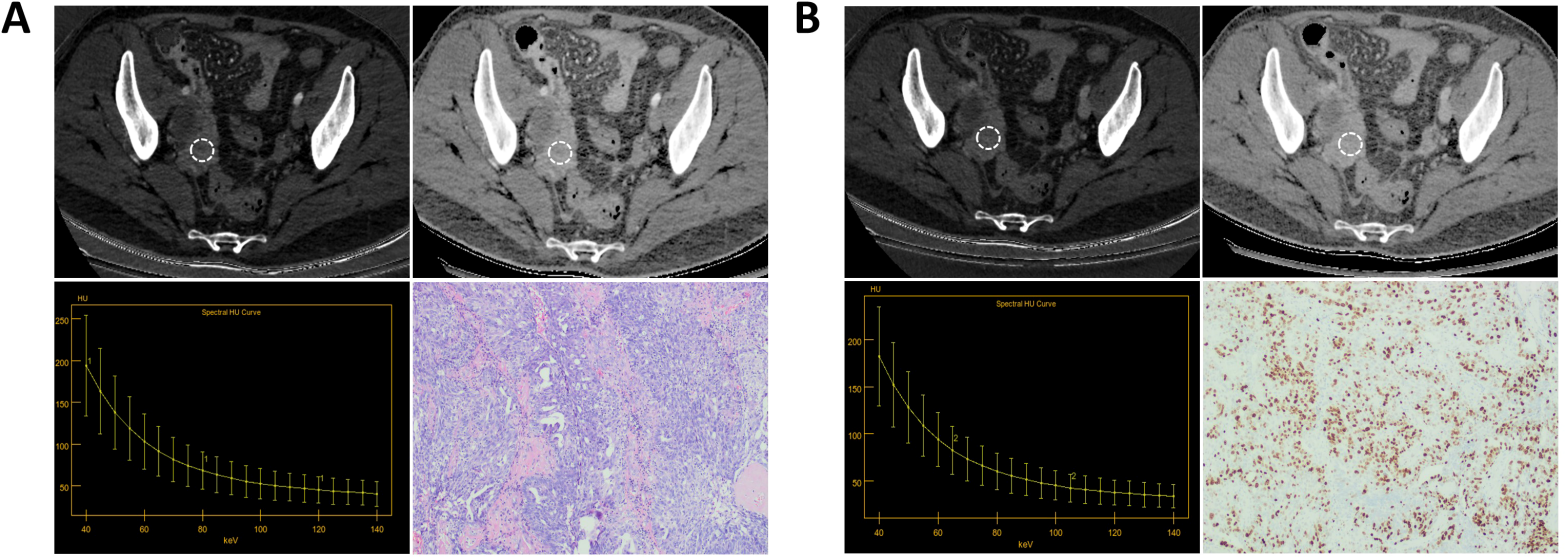
A patient with Ki-67 expression level of 70%. **(A)** The AP IC value = 17.50×100μg/cm^3^; The AP Zeff value = 8.63; The AP λHU value = 3.11; HE staining showed high-grade serous carcinoma of the right ovary (magnification, 100x). **(B)** The VP IC value = 16.13×100μg/cm^3^; The VP Zeff value = 8.57; The VP λHU value = 3.17; Immunohistochemistry shows Ki-67 positivity of about 70% (magnification, 100x).

**Figure 5:**
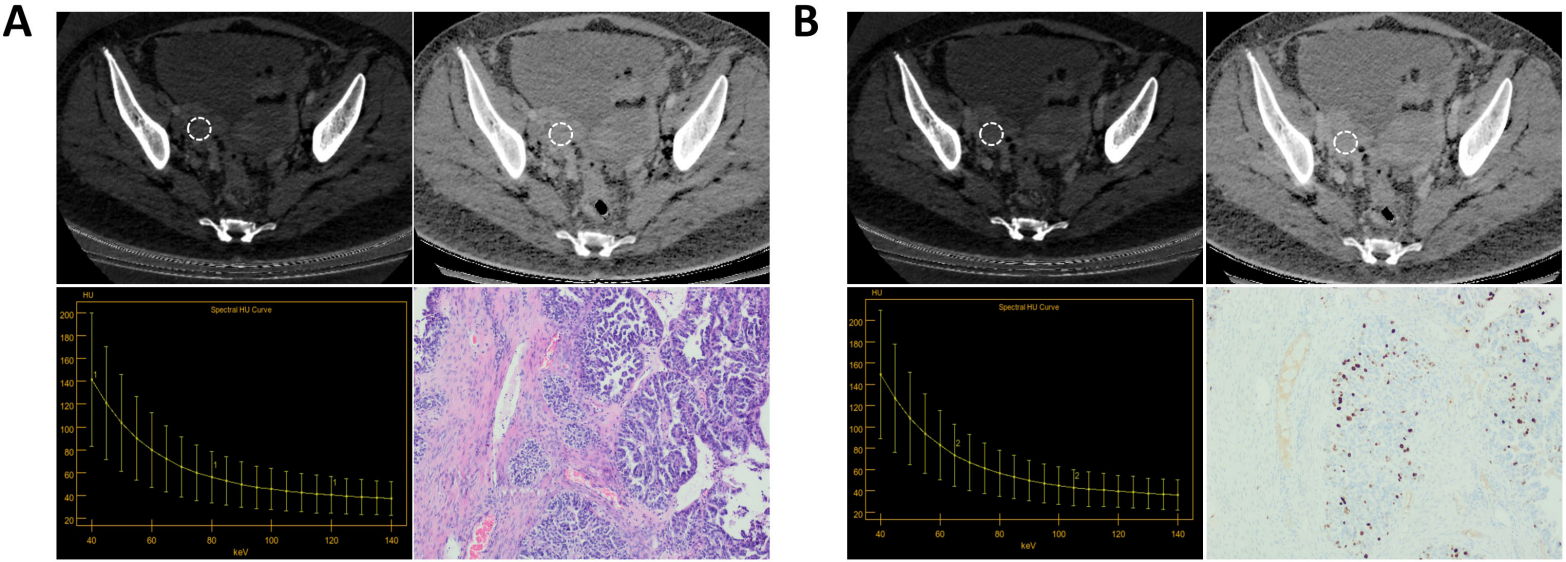
A patient with Ki-67 expression level of 40%. **(A)** The AP IC value = 12.13×100μg/cm^3^; The AP Zeff value = 8.34; The AP λHU value=2.08; HE staining showed high-grade serous carcinoma of the right ovary (magnification, 100x). **(B)** The VP IC value = 10.95×100μg/cm^3^; The VP Zeff value = 8.26; The VP λHU value =2.30; Immunohistochemistry shows Ki-67 positivity of about 40% (magnification, 100x).

**Figure 3** and **Table 3** show that the spectral CT parameters, including A-sIC, A-sZeff, V-IC, D-Zeff, and D-sZeff, were considerably distinct between the two groups, with the Ki-67 high-level group displaying higher values (mean A-sIC,0.52 ± 0.61vs.0.22 ± 0.23; mean A-sZeff, 0.87 ± 0.16 vs.0.66 ± 0.25; mean V-IC,19.59 ± 13.72 (100μg/cm^3^) vs.13.01 ± 8.08 (100μg/cm^3^); mean D-Zeff,8.78 ± 0.84 vs.8.23 ± 0.68; mean D-sZeff,1.08 ± 0.37 vs.0.81 ± 0.28; *P*<0.05). In addition, for A-IC, A-Zeff, A-λHU, V-sIC, V-Zeff, V-sZeff, V-λHU, D-IC, D-sIC, and D-λHU, the Ki-67 high-level group exhibited somewhat higher values than the low-level group in all three phases. However, all P values were more than 0.05, suggesting that the variation did not approach statistical relevance. **Figures 4** and **5** respectively depict the examples with low and high Ki-67 levels.

**Table 3:** Comparative analysis of two groups’ spectral CT parameters.

Parameters	Ki-67 high-level (n=29)	Ki-67 low-level (n=23)	*P* value
A-IC (100μg/cm^3^)	18.18 ± 10.38	13.11 ± 8.26	0.4
A-sIC	0.52 ± 0.61	0.22 ± 0.23	0.028*
A-Zeff	8.60 ± 0.39	8.41 ± 0.39	0.1
A-sZeff	0.87 ± 0.16	0.66 ± 0.25	<0.001*
A-λHU	2.83 ± 1.43	2.18 ± 1.24	0.53
V-IC (100μg/cm^3^)	19.59 ± 13.72	13.01 ± 8.08	0.047*
V-sIC	0.48 ± 0.23	0.37 ± 0.16	0.058
V-Zeff	8.59 ± 0.38	8.43 ± 0.37	0.14
V-sZeff	0.96 ± 0.22	0.89 ± 0.05	0.164
V-λHU	2.83 ± 1.46	2.23 ± 1.15	0.109
D-IC (100μg/cm^3^)	18.15 ± 9.31	13.87 ± 7.37	0.078
D-sIC	0.71 ± 0.39	0.59 ± 0.34	0.246
D-Zeff	8.78 ± 0.84	8.23 ± 0.68	0.014*
D-sZeff	1.08 ± 0.37	0.81 ± 0.28	0.006*
D-λHU	2.91 ± 1.50	2.28 ± 1.16	0.103

IC, Iodine concentration; sIC, standardized iodine concentration; Zeff, effective atomic number; sZeff, standardized effective atomic number; λHU, The slope of the spectral curve; A, Arterial phase; V, Venous phase; D, Delayed phase. * indicates the significant difference between two parameters.

### 3.4 Correlation analysis between spectral CT parameters and Ki-67 levels

**Table 4** shows the correlation coefficients and 95% confidence intervals. The data disclosed a positive correlation between A-IC, A-sIC, A-Zeff, A-sZeff, A-λHU, D-IC, D-Zeff, and D-sZeff with Ki-67 status, with correlation coefficients of 0.301, 0.354, 0.290, 0.417, 0.292, 0.277, 0.284, and 0.301, respectively, all *P* values < 0.05. Among them, the correlation between A-sZeff and Ki-67 levels was the strongest (r = 0.417), showing a moderate positive correlation, while the other parameters revealed a weak positive correlation(correlation criteria: strong correlation: 0.7 <|r|< 1; moderate correlation: 0.4 <|r|< 0.7;weak correlation: 0 <|r|< 0.4).

**Table 4:** Correlation between spectral CT parameters and Ki-67 levels.

Parameters	r(95%CI)	*P* value
A-IC	0.301(0.022,0.536)	0.030*
A-sIC	0.354(0.081,0.577)	0.010*
A-Zeff	0.290(0.011,0.528)	0.037*
A-sZeff	0.417(0.154,0.624)	0.002*
A-λHU	0.292(0.012,0.529)	0.036*
V-IC	0.270(-0.012,0.511)	0.053
V-sIC	0.220(-0.065,0.471)	0.118
V-Zeff	0.223(-0.061,0.474)	0.112
V-sZeff	0.095(-0.191,0.365)	0.505
V-λHU	0.258(-0.024,0.502)	0.065
D-IC	0.277(-0.003,0.518)	0.047*
D-sIC	0.218(-0.067,0.470)	0.12
D-Zeff	0.284(0.004,0.523)	0.041*
D-sZeff	0.301(0.023,0.537)	0.030*
D-λHU	0.262(-0.020,0.505)	0.061

IC, Iodine concentration; sIC, standardized iodine concentration; Zeff, effective atomic number; sZeff, standardized effective atomic number; λHU, The slope of the spectral curve; A, Arterial phase; V, Venous phase; D, Delayed phase; 95%CI: 95% Confidence interval. * indicates the significant difference between two parameters.

### 3.5 The diagnostic efficacy of spectral CT parameters

**Figure 6:**
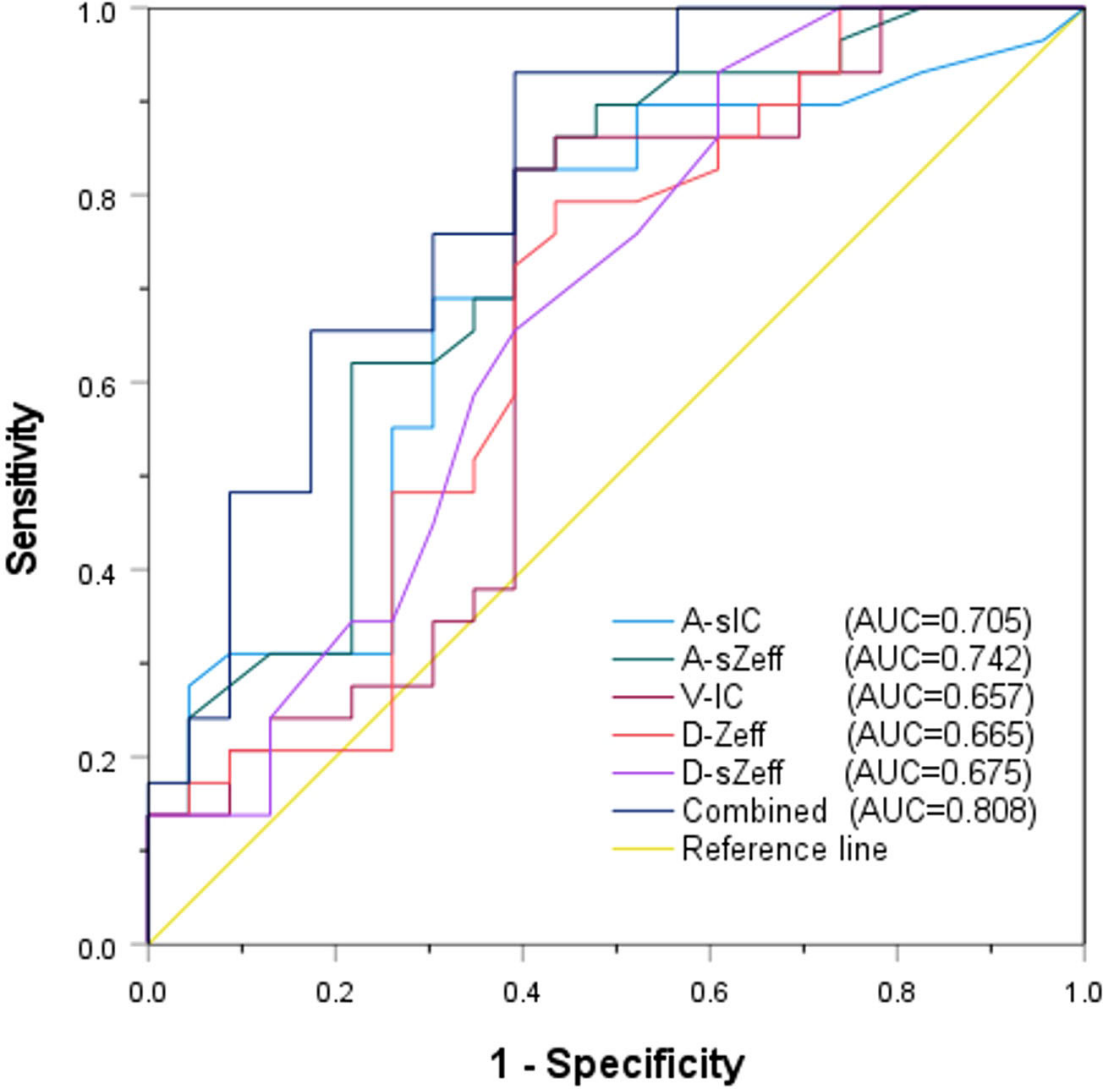
ROC analysis of relevant spectral CT parameters.

The diagnostic potential of a number of significant spectral CT characteristics is shown in **Table 5** and **Figure 6**. For A-sIC, A-sZeff, V-IC, D-Zeff, and D-sZeff, the corresponding AUCs were 0.705, 0.742, 0.657, 0.665, and 0.675. For predicting the Ki-67 status of ovarian cancer, A-sZeff revealed the highest diagnostic efficiency among the single spectral CT measures (AUC = 0.742, 95% CIs: [0.603-0.881], cutoff value = 0.765, sensitivity = 82.80%, specificity = 60.90%). Additionally, the combined model comprising A-sIC, A-sZeff, V-IC, D-Zeff, and D-sZeff showed remarkable efficacy in forecasting the level of Ki-67 of ovarian cancer by multivariate logistic regression (AUC = 0.808, 95% CIs: [0.687-0.929], cutoff value = 0.466, sensitivity = 93.10%, specificity = 60.90%). Through single-factor and multiple-factor logistic regression analysis, it was found that A-sZeff is an independent predictor of Ki-67. Besides, Delong’s test compared the AUC of the combined model with the other individual parameters (A-sIC, A-sZeff, V-IC, D-Zeff, D-sZeff) and showed that there were significant differences (all *P* values <0.05) between the combined model and other individual parameters, as well as between individual parameters. This result indicates good comparability between the models.

**Table 5:** Diagnostic performance of spectral CT parameters.

P9arameters	AUC (95%CI)	Cutoff value	Sensitivity (%)	Specificity (%)
A-sIC	0.705(0.559,0.851)	≥0.125	82.80%	60.90%
A-sZeff	0.742(0.603,0.881)	≥0.765	82.80%	60.90%
V-IC	0.657(0.498,0.815)	≥12.875	82.80%	60.90%
D-Zeff	0.665(0.511,0.819)	≥8.415	79.30%	56.50%
D-sZeff	0.675(0.523,0.826)	≥0.895	93.10%	39.10%
Combined	0.808(0.687,0.929)	≥0.466	93.10%	60.90%

IC, Iodine concentration; sIC, standardized iodine concentration; Zeff, effective atomic number; sZeff, standardized effective atomic number; A, Arterial phase; V, Venous phase; D, Delayed phase; AUC:

The area under the curve; 95%CI, 95% Confidence interval.

## 4 Discussion

Spectral CT, a novel imaging technique, offers several quantitative characteristics for illness diagnosis and analysis (23, 24). This study examined the relationship between multiple quantitative indicators obtained from spectral CT and Ki-67 levels in ovarian cancer. Our findings indicated that certain spectral CT parameters, such as IC and Zeff, were considerably elevated in the Ki-67 high-level group than in the low-level group. Moreover, in certain phases of IC, sIC, Zeff, sZeff, and λHU values were positively correlated with the Ki-67 level. However, the correlation between spectral parameters and Ki-67 levels is not strong, and the reason for this phenomenon is due to technical limitations or other reasons (such as a small sample size), which require a larger sample size for verification. By comparing the sensitivity and specificity of individual parameters and the combined parameter models, this study discovered that the optimal specificity is low, at roughly 60.9%, and the optimal sensitivity is high, at roughly 93.10%. This result indicates that the possibility of missed diagnosis is relatively small, and the possibility of misdiagnosis is relatively large. The model needs further improvement in the future to improve diagnostic efficiency. Although histology is the gold standard for identifying ovarian cancer, employing spectral CT to predict Ki67 may bring unexpected results to the diagnostic process.

The growth, maturation, and subsequent metastasis of tumor cells are greatly impacted by the blood vessels of tumors (25–27). IC possesses the ability to objectively display the blood flow status within the tumor in numerical form and quantitatively reflect the degree of vascular formation (28–30). Furthermore, NIC has the potential to lessen individual hemodynamic disparities, thereby enhancing the comparability of individuals (31). The A-sIC and V-IC values of the high-level group were markedly greater than those of the low-level group in this study. This finding is in alignment with the reality that cells with high Ki-67 levels exhibit a higher degree of proliferation and are accompanied by denser and more abundant angiogenesis (32, 33).

The effective atomic number of a substance, denoted by Zeff, can also serve as an indicator of the composition of substances within the tumor (34). Substantial distinctions were identified in Zeff between the two groups in this study, with increased Ki-67 levels corresponding to higher values. Furthermore, Zeff displayed a positive correlation with Ki-67 level, which is in line with certain findings from prior research (35, 36). The potential explanation is that cells with elevated Ki-67 expression levels show a greater degree of proliferation, which leads to a more compact arrangement of cells.

Diverse chemical molecules generate diverse energy attenuation curves, and different substances reflect varied chemical molecular structures. Consequently, according to the slope of the spectral curve, the disparity is quantifiable. The chemical compositions of various substances can be ascertained by comparing the inclinations of their spectrum curves (37, 38). However, in this study, we discovered that the λHU failed to vary significantly between two groups, despite the fact that there was a slight difference (*P* > 0.05). In contrast, Wang et al. (35)observed that the λHU value was considerably greater in the Ki-67 high-level group than in the low-level group. We believe that this variation is owing to the study’s low sample size, as well as the various degrees of tumor differentiation. More research is needed to figure out the efficiency of λHU by expanding the sample size and refining the differentiation degree.

In analyzing the diagnostic efficacy of spectral CT parameters for differentiating Ki-67 levels, ROC analysis findings suggested that the combined model behaved excellently, with relatively high sensitivity and specificity, whereas the univariate model performed moderately. As a result, spectral CT-derived parameters have a high predictive value for Ki-67 level in ovarian cancer, which is critical for predicting prognosis and guiding treatment of ovarian cancer patients.

The study is restricted to specific constraints. Initially, it is retrospective, and selection bias may influence the outcomes. Second, the association between factors may be exaggerated due to the limited sample size utilized in this study, necessitating the inclusion of more participants to corroborate the study’s conclusions. Third, the pathogenic categories and differentiation degrees of ovarian cancers have not been grouped, and more research is needed to substantiate our preliminary conclusions. Additionally, this study is a single-center design and homogeneous patient population, which may limit external validity. Finally, the manual delineation of ROI in this study may have a certain impact on the experimental results, and AI-driven segmentation can be used as an alternative in the future to improve reproducibility.

In conclusion, the level of Ki-67 in ovarian cancer can be predicted using the quantitative characteristics of spectral CT. When it came to differentiating Ki-67 level in ovarian cancer, the multivariate model including spectral parameters performed better diagnostically than the univariate model. Spectral CT-derived IC and Zeff could be helpful metrics for assessing the degree of Ki-67 expression in ovarian cancer. The combined model demonstrated a good diagnostic performance (AUC=0.808), and spectral CT is expected to replace or complement biopsy for Ki-67 assessment in resource-limited settings.

## Data Availability

The original contributions presented in the study are included in the article/supplementary material. Further inquiries can be directed to the corresponding author/s.
